# Gene Conversion in Angiosperm Genomes with an Emphasis on Genes Duplicated by Polyploidization

**DOI:** 10.3390/genes2010001

**Published:** 2011-01-10

**Authors:** Xi-Yin Wang, Andrew H. Paterson

**Affiliations:** 1 Plant Genome Mapping Laboratory, University of Georgia, Athens, GA 30602, USA; 2 Center for Genomics and Computational Biology, School of Life Sciences and School of Sciences, Hebei United University, Tangshan, Hebei 063009, China

**Keywords:** Non-allelic (ectopic) gene conversion, gene duplication, illegitimate recombination, angiosperm, grass

## Abstract

Angiosperm genomes differ from those of mammals by extensive and recursive polyploidizations. The resulting gene duplication provides opportunities both for genetic innovation, and for concerted evolution. Though most genes may escape conversion by their homologs, concerted evolution of duplicated genes can last for millions of years or longer after their origin. Indeed, paralogous genes on two rice chromosomes duplicated an estimated 60–70 million years ago have experienced gene conversion in the past 400,000 years. Gene conversion preserves similarity of paralogous genes, but appears to accelerate their divergence from orthologous genes in other species. The mutagenic nature of recombination coupled with the buffering effect provided by gene redundancy, may facilitate the evolution of novel alleles that confer functional innovations while insulating biological fitness of affected plants. A mixed evolutionary model, characterized by a primary birth-and-death process and occasional homoeologous recombination and gene conversion, may best explain the evolution of multigene families.

## 1. Introduction

### 1.1. Gene Duplication in Angiosperms

One characteristic distinguishing angiosperm genomes from most animal genomes is wide-spread and recursive whole-genome doubling (or tripling) events, which makes angiosperm genomes more complex in structure and DNA content [1]. Despite that the plant genomes that have been sequenced to date are smaller than average among angiosperms, all had a polyploid ancestor. For example, most if not all eudicots have a hexaploid ancestor [1,2], with the ancestors of *Arabidopsis*, poplar (*Populus trichocarpa*), cotton (*Gossypium*) and soybean (*Glycine max*) all affected by 1–2 additional whole-genome doublings [3–7]. While it remains unclear if the eudicot paleo-hexaploidy affected monocots, the common ancestor of grasses was affected by at least two whole-genome doublings [8–11]. These large-scale events each produced tens of thousands of duplicated genes per genome, and some remain in their ancestral gene orders in large duplicated regions, termed to be homoeologous to one another, that are still discernible. They may change an organism's evolutionary trajectory over-night, vividly exemplifying the notion of punctuational change in evolution. However, the impact of genome doubling is also long-lasting, sometimes noted to be a two-phase process [12]. Though dramatic changes in a genome may often occur soon after a polyploidization event, such as chromosomal segmental reshuffling and gene losses [3,8,13,14], duplicated genomes and gene sets continue to provide for functional innovation over millions of years (my). Transposable element-related and other single-gene events may also contribute to the production of duplicated genes, especially in angiosperms (which we will generally refer to herein as “plants”).

Events that expand the size of gene families provide opportunities for a variety of evolutionary changes to occur. It has long been realized that gene redundancy may allow relatively free changes in gene sequences, with a nucleotide mutation in one copy of a duplicated gene functionally buffered by the presence of other copies, mitigating the effect of the mutation on biological fitness. Some mutations may confer novel functions (neofunctionalization), or subdivision of ancestral functions (subfunctionalization), or a mixture of both (subneofunctionalization) [15–17]. On the other hand, duplicated genes may also interact directly through sequence contact and exchange, *i.e.*, DNA recombination. However, though recombination plays a central role in plant biology, possibly being higher and more variable than in animals [18], its effect on plant evolution may yet remain underappreciated and our knowledge of recombination rates and patterns in plants is far from comprehensive.

### 1.2. Genetic Recombination and Gene Conversion

As a driving force of biological evolution, genetic recombination is important for DNA repair and crossing-over between homologous sequences. New alleles and combinations of alleles produced by recombination may permit adaptation to environmental changes [19]. During meiosis, homologous chromosomes may recombine reciprocally, while during mitosis in somatic cells recombination can be induced by DNA damage. However, recombination, especially “illegitimate” recombination between paralogous genes, those originated in DNA duplication events, may produce severe chromosomal lesions characterized by various DNA rearrangements which are often deleterious and may cause severe human diseases [20], but may also contribute to elimination of deleterious mutations [21]. In plants, both meiotic and mitotic recombination outcomes can be transferred to the offspring, due to the lack of a predetermined germline.

Genome duplication (polyploidization) may create conditions that are especially conducive to “illegitimate” recombination between non-homologous sequences. Duplicated genes produced by polyploidization often remain in ancestral chromosomal locations, with large-scale gene collinearity facilitating homoeologous recombination. Recombination between duplicated genes at ectopic locations may be facilitated by proximity, or by appreciable sequence similarity in their respective vicinities, *i.e.*, each being located among multiple members of the same gene family. Paralogous recombination can be reciprocal, with symmetrical exchange of genetic information between paralogous loci; or nonreciprocal, with unidirectional transfer of information from one locus to another, resulting in gene conversion [22]. Gene conversion was initially used to explain aberrant segregation ratios (6:2 and 5:3) other than the normal ratio (4:4) between two alleles at the same locus during meiosis in pink bread mold (*Neurospora crassa*) [23]. Gene conversion can typically be explained by DNA double-strand breaks that trigger DNA exchange between the homologous/homoeologous strands [24,25]. Here, we review recent findings about gene conversion in plants, especially on a genomic scale with emphasis on genes duplicated by polyploidization. We note that gene conversion through illegitimate recombination may have played a crucial role in plant genome evolution.

## 2. Gene Conversion between Duplicated Genes Produced by Polyploidization

### 2.1. Gene Conversion between Duplicated Genes Produced by Ancient Polyploidies

**Figure 1:**
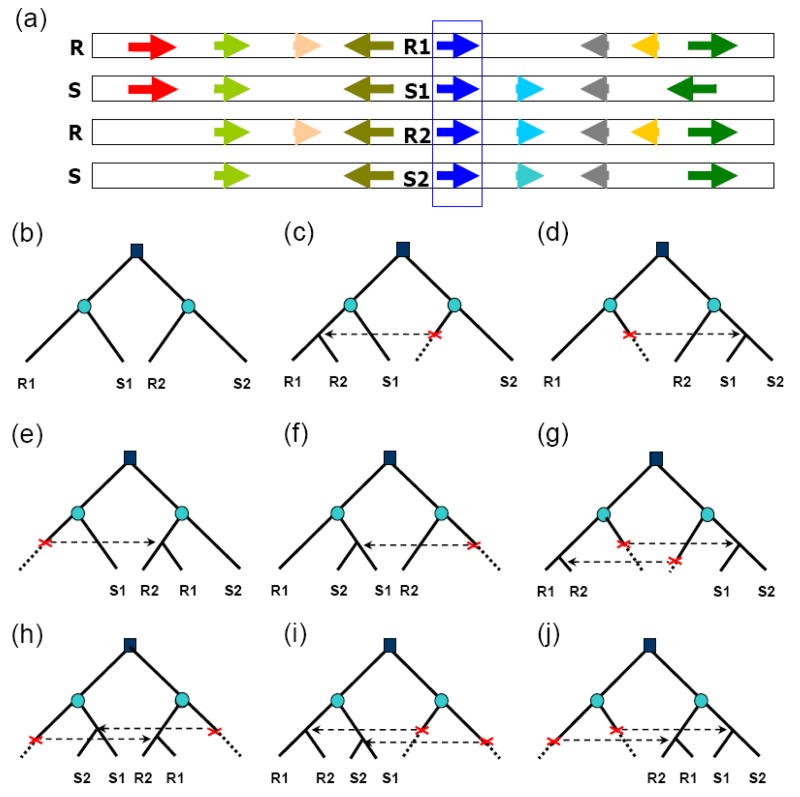
Definition of homologous gene quartets and inference of conversion based on phylogenetic topology changes. In (**a**), arrows show genes with colors reflecting homology. Gene quartets are formed by rice (R) paralogs R1 and R2, and their respective sorghum (S) orthologs S1 and S2. In (**b**) to (**j**), squares symbolize a duplication event in the common ancestral genome, and circles symbolize species divergence. The expected phylogenetic relationship of the homologous quartets is displayed in (**b**) if no conversion has occurred; (**c**) to (**j**) show different types of conversion, for example, (**c**) shows the expected pattern if gene R2 is converted by R1; (**d**) if gene S1 is converted by S2; and (**g**) if both the above conversions occurred.

Recent studies revealed appreciable rates of gene conversion between paleologous genes, those duplicated during ancient polyploidization. The availability of whole-genome sequences of rice (*Oryza sativa*) and sorghum (*Sorghum bicolor*) [26–28] provided for genome-wide inference of gene conversion using a comparative genomics method. About 20% of duplicated genes resulting from paleopolyploidy have been preserved in the modern rice and sorghum genomes [8,9,29], and not less than 97% of these are preserved in both species. By identifying aberrant tree topology change within “quartets” of duplicated genes in rice and sorghum (Figure 1), about 14% of rice duplicates and 12% of sorghum duplicates were found to have been affected by gene conversion after the divergence of the two lineages [30]. About 40% of converted genes showed evidence of conversion along their entire length, and 60% along only part of their length. Since the two lineages diverged an estimated 50 mya [31], their common ancestor had experienced roughly 20 million years of evolution following the whole-genome duplication, during which time the ancestral polyploid genome might have largely regained structural stability and disomic pairing (for example, as reflected by 80% loss of duplicated genes). Nonetheless, many duplicated genes continued to evolve in concert to at least some degree, and indeed appear to be still doing so today in some parts of the rice genome. Gene conversion has been shown to have occurred in the last 0.4 my [32], after the divergence of two *Oryza sativa* subspecies *indica* [26] and *japonica* [27,33]. Since divergence from subspecies *indica*, ∼8% of *japonica* paralogs on chromosomes 11 and 12 have been affected by gene conversion and reciprocal exchanges of chromosomal segments. Functional domain-encoding sequences are more frequently converted than nondomain sequences.

Cotton (*Gossypium*) allopolyploid genomes (AD) originated 1–2 mya [6]. By utilizing cotton EST sequences to identify homoeologous single nucleotide polymorphisms, recombination regions displaying two recombination breakpoints have been inferred, ranging between 1 and 698 nucleotides in length [34]. The authors extrapolated that gene conversion may have modified 1.8% of the cotton transcriptome. There was evidence supporting both reciprocal homoeologous recombinations or crossing-overs (0.4%), and gene conversion affecting both A and D copies but showing different conversion patterns (0.2%). The authors verified 14 of 20 selected cases of putative gene conversion events from genomic DNA by Sanger sequencing. The cotton gene conversion rate seems to be lower than that in grasses but may be underestimated due to the use of EST assemblies rather than genomic DNA sequence.

### 2.2. Gene Conversion and Crossing-Over Contribute to Singular Evolution of Two Grass Chromosomes

**Figure 2:**
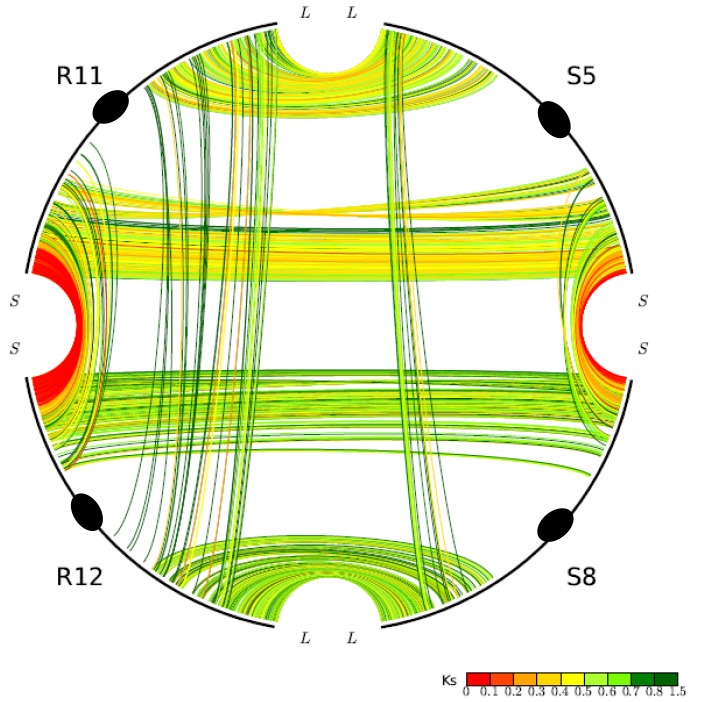
Homology pattern of chromosomes rice chromosomes R11, R12, and their respective sorghum orthologs S5 and S8. Chromosomes are shown with black curved lines, with ovals displaying centromeres. “L” and “S” show long and short arms of the chromosomes. The interior lines show the duplicated genes within genomes and orthologs between genomes. Colors of lines show Ks values (synonymous substitution rates between homologous genes), as illustrated in the right-bottom corner.

One unusual duplicated genomic region in grasses has been subject to a remarkably high level of concerted gene evolution. Previously, it was suggested that rice chromosomes 11 and 12 share a segmental duplication near the termini of the short arms, dated to only 5–7 mya by various groups [8,26,35]. However, there have been suspicions about the date and origin of the duplicated segments, particularly based on the observation that no homoeologs from the 70-mya whole-genome doubling event could be identified [9]. With the availability of the sorghum genome sequence, a similar duplicated segment also appearing much younger than the 70-mya duplication was found on sorghum chromosomes 5 and 8, orthologous to rice chromosomes 11 and 12 (Figure 2). (Note: Homologous sequences are orthologous if they were separated by a speciation event). It seems prohibitively unlikely that two independent lineages would each experience recent segmental duplications in corresponding regions of one and only one pair of paleo-duplicated chromosomes. Much more probable is our alternative hypothesis that the region was not a pair of segmental duplications, but resulted from the pan-cereal whole-genome duplication and became differentiated from the remainder of the genome due to concerted evolution acting independently in sorghum, rice, and probably additional cereals. This hypothesis is strongly supported by an analysis of *intra*- and *inter*-species syntenic genes. While sorghum-sorghum and rice-rice paralogs from this region show Ks values of 0.44 and 0.22, respectively, sorghum-rice orthologs show Ks of 0.63, which seems to preclude the possibility of species-specific duplications but is very consistent with concerted evolution in these regions since the rice-sorghum split. “Parallel concerted evolution” may have also occurred in corresponding regions of other cereals. Indeed, physical and genetic maps suggest shared terminal segments of the corresponding chromosomes in wheat (*Triticum aestivum*, 4 and 5) [36], foxtail millet (*Setaria italica*, VII and VIII), and pearl millet (*Pennisetum glaucum*, linkage groups 1 and 4) [37].

### 2.3. Chromosomal Changes and Suppression of Homoeologous Recombination

The availability of whole genome sequence helps considerably toward understanding how genome changes have affected the occurrence of gene conversion. Despite having occurred millions of years ago, whole-genome duplication events have resulted in extensive changes to genes and chromosomes that are still discernible in many extant genomes. Scrutiny of ancient polyploidizations provides valuable information of the long-term genome-wide consequences of concerted evolution, while resynthesized polyploids let us have a close look at the underlying mechanisms in action.

Gene conversion is unevenly distributed through the rice and sorghum genomes. The most affected chromosomes are rice chromosomes 11 and 12 and their sorghum orthologs 5 and 8, respectively, in which about 60% of syntenic genes have been affected [30]. At one end of rice chromosomes 11 and 12, tens of duplicated genes are experiencing on-going concerted evolution and remain nearly identical in sequence [32]. The orthologous rice and sorghum chromosomes are similar in gene conversion rates.

While the exact properties that have led to the unusual concerted evolution of rice chromosomes 11 and 12 and their sorghum orthologs remain elusive, the general long-term maintenance of concerted evolution might be explained as follows. Genome doubling is often followed by genome instability, characterized by massive DNA rearrangements, inversions and DNA losses, eventually re-establishing diploid heredity. Soon after polyploidization, multiple homologous chromosomes or chromosomal segments may compete to pair and recombine with one another, forming multivalent structures during meiosis. DNA rearrangement may inhibit pairing between affected chromosomes or chromosomal segments. Gradually, structural and sequence divergence may lead to the formation of neo-homologous chromosome pairs that preferentially pair with one another during meiosis. Those chromosomes or chromosomal segments sharing ancestry, but which are sufficiently diverged from one another in structure and sequence to inhibit pairing, are then referred to as homoeologous. Thereafter, though wide-spread and frequent recombination between homoeologous DNA segments may have been restricted, occasional and small-scale recombination may persist for a long time.

The sizes of duplicated blocks of genes are positively correlated with gene conversion rates. The smallest blocks have the least conversion. When small duplicated blocks are buried in chromosomes that otherwise share little homoeology, they may have little chance to pair. This may be particularly true when other regions of the chromosome have large-scale homoeology with other chromosomes, leaving the small duplicated regions at a disadvantage in forming homoeologous duplexes.

DNA inversion may have directly contributed to recombination restriction between homoeologous regions. Though rice chromosomes 1 and 5 share large-scale homoeology characterized by ∼600 homoeologous genes, the conversion rate between them is as low as 3.4%, as is also the case with the orthologous sorghum chromosomes. One possible explanation is that the homoeologous genes are in two isolated groups near each end of the chromosomes, and that a large inversion in the short arm before rice-sorghum divergence [28] may have reduced competence to form homoeologous duplexes.

Recombination might have been restricted in different genomic regions in a non-synchronized manner. Conversion rates differ among duplicated blocks in rice and sorghum. One factor that may contribute to this variation is that anti-recombination factors such as chromosomal rearrangements might have occurred in a stochastic way among homoeologous blocks, *i.e.*, some may have been restricted earlier than others. Interestingly, the rice and sorghum orthologous chromosomes/chromosomal segments show similar patterns of gene conversion. This might be explained in that the divergence levels between the ancestral paralogous chromosomes in the cereal common ancestor influenced the recombination pattern in the offspring.

## 3. Gene Conversion Members in Large Gene Families

### 3.1. Models of Concerted or Divergent Evolution

Gene conversion has long been invoked to explain the evolution of large gene families [38,39], such as rRNA genes [40–42] and histone genes. Different lines of analysis showed that large gene families often evolved in a birth-and-death manner accompanied by strong purifying selection [43,44]. This helps the members in a family to be highly similar in sequence, contributing to the occurrence of gene conversion. The recombination that leads to gene conversion may incur nucleotide mutation as in the double-strand breakage model. Gene conversion tends to homogenize affected gene copies, and may, therefore, transfer mutations among gene copies, possibly leading to their evolution as a unit [45], referred to as concerted evolution. A program named GeneConv is often used to infer gene conversion in gene families, based on aligned segments for which a pair of sequences are unexpectedly similar [46].

Until around 1990, most multigene families were thought to be subject to concerted evolution through gene conversion and associated homogenization of gene sequences [45]. This seems true for rRNA and histone gene families and some other highly conservative ones, for all the units are often highly similar. However, as reported previously, this seems not completely true for other multigene families. The evolution of some families may be better explained by a birth-and-death model [45] in which new genes are created by gene duplication and some genes stay in the genome for a long time, whereas others are inactivated or deleted. Here, by reviewing evolution of several multigene families, especially disease resistance gene families and rRNA gene families, we emphasize a mixed model of birth-and-death and gene conversion as a general model explaining the evolution of multigene families, as further discussed later in this section.

### 3.2. Genome-Wide Search for Concerted Evolution of Multigene Families

Xu *et al.* performed a genome-wide inference of gene conversion in 626 rice multigene families, in which they detected 377 gene conversion events [47]. Over 60% of conversions were detected between specific chromosome pairs, particularly chromosomes 1 and 5, 2 and 6, and 3 and 5. The first two pairs were produced by the 70-mya whole-genome duplication, indicating that paleologous gene pairs may account for an appreciable percentage of their inferred converted genes. However, using the same approach, another group failed to find any evidence of gene conversion between *Arabidopsis thaliana* paleologs produced by whole-genome duplication [48]. This incongruity may result from the fact that Arabidopsis genes diverge much faster than rice genes [1] and therefore may more quickly escape conversion and restore independent evolutionary paths.

### 3.3. Gene Conversion and Evolution of Disease Resistance Genes

Plant disease resistance genes form large gene families and are frequently clustered in the genome [49], conditions that may favor gene conversion and have stimulated much research into whether they frequently experience ectopic recombination. Genes for resistance (R-genes) to diverse pathogens cloned from several species encode proteins with common motifs which are part of certain signal-transduction systems. Most of these R-genes encode a leucine-rich repeat (LRR) region. Mondragon *et al.* described the extent and characteristics of gene conversion and unequal crossing-over in the coding and noncoding regions of nucleotide-binding site leucine-rich repeat proteins (NBS-LRR), receptor-like kinases (RLK), and receptor-like proteins (RLP) in the plant *Arabidopsis thaliana* [50]. The authors found the occurrence of gene conversion to be significantly associated with high levels of sequence similarity, close physical proximity (clustering), gene orientation, and recombination rate. Study of recombination and spontaneous mutation events within clusters of resistance gene *Dm* in lettuce for multiple generations identified sixteen mutants, corresponding to mutation rates of 10^−3^ to 10^−4^ per generation [51]. DNA deletion events were associated with exchange of flanking markers, consistent with unequal crossing over. They noted that one mutant was the result of a gene conversion event between *Dm3* and a closely related homolog, generating a novel chimeric gene. The authors further showed that spontaneous deletions were correlated with elevated levels of recombination. Sun *et al.* analyzed the evolution of Rp1, a complex rust resistance locus of maize and eight paralogs, seven of which code for predicted NBS-LRR proteins similar to the Rp1-D gene [52]. The authors found no evidence of gene conversion but noted that crossing-overs led to reduced resistance specificity of the Rp1-D gene.

The above examples show that gene conversion may have occurred during the evolution of plant disease resistance genes but may not be the primary factor driving the evolution of R-genes. In particular, the LRR regions, assumed to mediate host-pathogen interaction, are hypervariable and have elevated ratios of nonsynonymous to synonymous substitutions [49]. Previously, generation of new resistance specificities had been thought to involve frequent unequal crossing-over and gene conversions. However, comparisons between resistance haplotypes reveal that orthologous genes are more similar than paralogous genes, implying a low rate of sequence homogenization. Genome-wide survey indicates that there are a lot of young of R-genes forming clusters on chromosomes, but there are also always many highly diverged R-genes, indicating high mutation rates [53,54]. Therefore, a birth-and-death model emphasizing divergent selection acting on arrays of solvent-exposed residues in the LRR may better describe the evolution of R-genes than a concerted evolution model [49].

## 4. Factors Related to Gene Conversion

### 4.1. Conversion and Physical Location

The physical location of genes may correlate with their chance of being converted. Converted paleologs in rice and sorghum are often in distal regions of chromosomes [30]. In rice, affected genes have an average distance of 6.1 Mb to chromosome termini, with wholly converted being 3 Mb, as compared with an average of 6.6 Mb for the total set rice genes in “quartets” used to infer gene conversion. In sorghum, affected genes have an average distance of 7.6 Mb to termini, with wholly converted being 5.4 Mb, as compared with 8.6 Mb for genes in quartets. In rice, >50% of wholly converted genes are in the initial 2 Mb from the chromosomal termini, in which ∼40% of the duplicated genes have been converted. In sorghum, 48.6% of wholly converted genes are in the initial 2 Mb from the chromosomal termini, in which ∼34.5% of the duplicated genes have been converted.

Assuming that sequence similarity is the physical basis for recombination, a correlation of physical (terminal) location with gene conversion probability is supported by several lines of evidence. First, gene sequences, more conservative than other sequences, are often more abundant in distal chromosomal regions (away from centromeres) where their sequences might be better preserved. Gene collinearity is often found in gene-dense (enchromatic) regions where homologous recombination is active but not in gene-scarce (heterochromatic) regions [55]. Active recombination may preserve sequence similarity by removing deleterious mutations [56]. Second, repetitive elements are often enriched in pericentromeric regions, which reduce large-scale sequence similarity between homoeologous segments by inducing DNA rearrangements and mutations. In both rice and sorghum, LTR elements are substantially enriched in the pericentromeric regions, making up ∼50% of rice and ∼80% of sorghum pericentromeric DNA *versus* only 20–30% of DNA in the gene-dense regions [26,28]. In the initial 2 Mb DNA short arms of rice chromosomes 11 and 12, where conversion is the highest, only ∼15% of sequences are LTRs, as compared with an average ∼42% throughout the genome [57] The corresponding regions in sorghum show similarly low levels of LTRs. Third, the mechanics of chromosome pairing may contribute to the physical distribution of gene conversion. Homologous pairing in early meiotic prophase is accompanied by dynamic repositioning of chromosomes in the nucleus and formation of a cytological structure called the telomere bouquet, *i.e.*, chromosomes that are bundled at the telomere to form a bouquet-like arrangement [58,59]. Duplicated blocks near the telomeres may have a larger chance to pair with one another to form DNA heteroduplex for a longer time than other regions, facilitating gene conversion.

### 4.2. Can Genes Escape Conversion?

Sequence similarity is a key factor affecting occurrence of recombination. Sequence divergence may limit the frequency, length, and stability of early heteroduplex intermediates formed during recombination, dramatically reducing the recombination frequency [60]. In model organisms, much research has been performed to better understand how sequence divergence affects the frequency of recombination. Research with a reporter system in Arabidopsis indicated that, relative to homologous sequences, there was a 4- to 20-fold decrease in the recombination frequency in lines with constructs containing 0.5%–9% sequence divergence [61]. In maize (*Zea mays*), the bronze (*bz*) gene is a recombination hotspot, and analysis of meiotic recombination between heteroallelic pairs of *bz* mutations reveals both insertion mutation and sequence divergence to affect the distribution of intragenic recombination events [62]. Adjacent retrotransposons abruptly decrease recombination rates in the *bz* locus [63]. In seven tomato lines, recombination frequency at two adjacent intervals on chromosome 6 was characterized [64]. When the entire chromosomal arm of tomato (*Lycopersicon esculentum*) was replaced with either chromatin of *Lycopersicon pimpinellifolium*, a closely related species, or of *Lycopersicon peruvianum*, a more distant species, up to a six-fold decrease in recombination frequency was observed. In partial summary, even a small decrease in sequence similarity is likely to suppress recombination between homologous sequences in allelic or proximal regions. It is reasonable to anticipate that recombination between duplicated genes at ectopic locations may be even more sensitive to sequence variation.

Thus, duplicated genes may escape conversion through divergence. This inference is supported by findings from duplicated genes produced by ancient polyploidization. Duplicated chromosomal blocks have long runs of sequence similarity represented by gene collinearity. Shortly after polyploidization, duplicated sequences might be very similar or even nearly identical in autopolyploidy, facilitating gene conversion. However, the similarity may diminish with time. For example, in rice and sorghum, though gene conversion may have affected the evolution of hundreds of genes, the overall non-corrected synonymous nucleotide substitution rates between paralogs in each species are about ∼0.5 [30]. This implies a generally low frequency of gene conversion between syntenic genes, and that the paleologs in rice and sorghum have generally escaped from conversion. A search for gene conversion in Arabidopsis found no evidence, with paralogs having restored independent evolution [48], as noted above perhaps aided by elevated gene evolutionary rates in Arabidopsis [1].

Gene conversion itself may be a factor to restrict its further occurrence. For genes in large families, conversion may be much more likely between young proximal duplicated copies based merely on high sequence similarity. However, if paralogs are subject to ectopic recombination, which is noted to be mutagenic [65,66], it is a force for divergent evolution. Second, as expected by evolutionary theory, redundancy may buffer occasional mutations, and therefore be another force contributing to divergence of gene family members. Conversion in only part of a gene may increase functional redundancy. While recombination and gene conversion can help to purge deleterious mutations, however, natural selection based on DNA repair mechanisms may not be efficient enough to remove mutations that are only mildly deleterious. It is, therefore, likely that most genes (excepting unusual cases such as on rice chromosomes 11,12 and sorghum 5–8) gradually accumulate a few mutations that begin to suppress further recombinations.

### 4.3. Concerted Evolution and Gene Birth and Death

It had long been proposed that concerted evolution may explain the evolution of rRNA genes, but other gene families seem to be better explained by the birth-and-death model [67]. However, even with rRNA genes, recently the role of concerted evolution has been challenged by the discovery of significant variation in yeast rRNA gene sequences within individual genomes [68]. Comparative analysis of the complete data set from different yeast genomes revealed that they possess different patterns of rRNA gene polymorphism, especially in the intergenic spacer region. Based on this research, it seems the rRNA genes may also better explained by a birth-and-death model. Therefore, the two forces that lead to divergent evolution pointed out above are widespread.

Homoeologous recombination and gene conversion may be somewhat circular, occurring most frequently (and conferring homogeneity) when duplicated genes are young and in proximal locations or in large duplicated regions, and becoming progressively less frequent as the sequences diverge. This has been clearly shown in the 70 mya paleologs in rice and sorghum. A mixed model, a primary birth-and-death process with occasional homoeologous recombination between duplicated genes, may best explain the evolution of multigene families.

The unusual paleologs on rice chromosomes 11 and 12, and their respective orthologs, sorghum chromosomes 5 and 8, seem to depart from the thesis that most duplicated genes gradually escape homogenizing forces and establish independent evolution. Some duplicated genes in these regions, especially those near the chromosomal termini, have been locked in continual *intra*-genomic homogenization for tens of millions of years after doubling [30,32]. Indeed, paralogs near the termini of the short arms of rice chromosomes 11 and 12 share nearly identical gene and intergenic sequences. That suggests that terminal paralogous regions on these chromosomes more than 100 Kb in length have recombined with one another much like homologous chromosomes. A terminal location alone may favor homoeologous recombination due to the mechanics of chromosome pairing. For homologous chromosomes, pairing in early meiotic prophase is accompanied by dynamic repositioning of chromosomes in the nucleus and formation of a cytological structure called the telomere bouquet, *i.e.*, chromosomes that are bundled at the telomere to form a bouquet-like arrangement [58,59]. The formation of the telomere clustering bouquet is closely related to the simple repeating sequences constituting the telomeres [58]. We hypothesize that the duplicated chromosomes have preserved the ancestral telomeres and the proximal chromosomal regions, and since whole-genome doubling, the telomeres and the neighboring regions have been preserved by frequent recombination. While recombinations may be far more frequently homologous than homoeologous, even a modest rate of homoeologous recombination may preserve high homoeologous DNA similarity. However, we cannot yet explain why the termini of other duplicated rice, sorghum, or other chromosomes do not exhibit this unusual behavior. To our knowledge, the exceptionally high rate of homoeologous recombination and gene conversion on rice chromosomes 11 and 12, and their respective orthologs, sorghum chromosomes 5 and 8, has not been found in any other species.

### 4.4. Genetic Control of Homoeologous Recombination

Since chromosomal pairing control and fertility are often related, it is assumed that fertile allopolyploids must have either had some level of pre-existing control over pairing, or in some way acquired this control during their evolution. Furthermore, suppression of homoeologous recombination is required to ensure proper chromosome pairing and segregation. Otherwise, complex meiotic configurations would lead to unbalanced gametes, aneuploid progenies, and, impaired fertility [69].

It is clear that homologous *versus* homoeologous chromosomal pairing is under genetic control. In the well-studied allohexaploid bread wheat (*Triticum aestivum*), three distinct, yet related genomes coordinate meiotic pairing such that all three sets of chromosomes (A, B and D) pair faithfully with their homologs and segregate disomically. Genetic control of chromosome pairing is mediated by the *PH1* locus [70–75]. Mutations at this locus lead to gross chromosomal rearrangements, and homoeologous recombination [73,76,77]. The *PH1* locus has been delineated to a 70 Mb region of chromosome 5B; however in more than 50 years since its first discovery, we are only starting to understand the mechanism by which it acts.

Similarly, in *Brassica napus* polyploids the *PrBn* locus regulates chromosome pairing, although its effect is only observed strongly at the allohaploid and allotriploid levels [78]. In cultured *B. napus* (AC) haploids, *PrBn*, localized on linkage group C9 within a 10–20 cM interval, is the main locus that determines the number of nonhomologous associations during meiosis of microspores. Nicolas *et al.* [79–82] examined the role played by *PrBn* in recombination by generating two haploid × euploid populations using two *B. napus* haploids with significantly different *PrBn* activity. The authors show that *PrBn* changes the rate of recombination between nonhomologous chromosomes during meiosis of *B. napus* haploids and also affects homologous recombination with an effect that depends on plant karyotype.

To our knowledge, it has been unclear whether these genetic controls of homoeologous recombination may restrict gene conversion, or whether it acts on ectopic or nonallelic recombination among clustered genes.

## 5. Gene Conversion and Genome Evolution

### 5.1. Gene Conversion Elevated Mutation Rates

Gene conversion has been suggested to explain low divergence rates between duplicated gene sequences found in several organisms [83,84]. Theoretically, gene conversion homogenizes paralogous gene sequences, whereas recombination that leads to gene conversion may contribute to divergent evolution. These forces may contribute to the evolution of gene families in opposite directions. Data regarding the evolution of large gene families has provided some support to this idea. However, the presence of very similar genes may make it difficult to distinguish young duplication from homogenization by conversion. What is difficult is that we must find a gene that have been credibly affected by gene conversion, and also prove that it has elevated evolutionary rate. Comparative analysis of duplicated genes from different species provides good support to the theoretical deduction, resolving this difficulty. According to two subsets of homologous quartets in rice and sorghum: one affected by conversion in both species, and the other not affected in either species [30], in each species the divergence (synonymous and nonsynonymous nucleotide substitutions) between converted paralogs are generally much smaller than those not converted, which indicates the effect of homogenization. In contrast, rice-sorghum orthologs in the conversion-affected subset have greater divergence than non-conversion-affected, showing that gene conversion has elevated evolutionary rates.

### 5.2. Gene Conversion and Functional Innovation

Though gene conversion keeps duplicated genes within the same nucleus similar to one another, *i.e.*, retards *intra*-genomic gene divergence, it is nonetheless a driving force of gene evolution. As repeatedly noted, recombination is a mutagenic factor, and mutations lay the foundation for natural selection.

First, as shown above gene conversion elevates evolutionary rates of affected genes, which drives the evolution of these converted genes.

Second, the ratio of nonsynonymous to synonymous nucleotide substitutions is often taken as an indicator of positive selection, though it is regarded as conservative [85]. The converted rice and sorghum paralogs have a higher ratio of nonsynonymous to synonymous nucleotide substitutions than non-converted paralogs, suggesting a significant selection pressure difference [30].

Third, gene conversion and/or related homoeologous recombination may directly affect gene function by replacing the functional genes' sequences. As reported, crossing-over led to reduced resistance specificity of the Rp1-D gene [52].

Fourth, in highly recombining regions, deleterious mutations tend to be removed and beneficial mutations may be fixed [18]. Syntenic genes are usually located in highly recombining regions, and gene conversion between them may remove deleterious but keep and transfer beneficial mutations. Theoretically, the converted regions on rice chromosomes 11 and 12 may have accumulated many beneficial mutations that may have contributed to the preservation of these regions and functional innovation in each lineage.

Fifth, homogenization by gene conversion may provide buffering that protects the evolution of functionally important genes and fixation of beneficial mutations. It is hypothesized that after polyploidy a primary advantage of retaining long complex genes is the buffering of crucial functions [84]. Gene conversion may homogenize partial or full gene sequences, which makes highly similar gene copies, constituting functional redundancy. Therefore, if new mutations occur in one of the copies, the other ones may execute the original function, consequently buffering the evolution of novel function in the first copy.

### 5.3. Gene Conversion and Evolutionary Analysis

To understand the evolutionary importance of a gene family, we often assume that the genes have been evolving independently, largely following some molecular clock, and a credible phylogeny can be reconstructed. These assumptions have been adopted in many molecular evolutionary theories and methods [86–88]. However, as noted above, when one gene is converted by another, their evolutionary trajectories are not independent. The transfer of information through conversion between them may constitute a leap forward or backward in evolution, possibly leading to aberrant phylogenetic tree topology that does not reflect the true evolutionary history of the gene family. If conversion events have been frequent, the tree phylogeny cannot be reconstructed. Up to now, consideration of gene conversion in analyzing the evolution of gene families has generally been inadequate, except for some valuable early explorations [89,90].

### 5.4. Homeologous Gene Conversion Does Not Cause the Elevation of Guanine and Cytosine (GC) Content in Grasses

Gene conversion has been linked by some to the elevation of GC content [91]. It has been hypothesized that conversion may be accompanied by DNA repair of nucleotide mismatches. If the repair process were biased towards G and C (referred to biased gene conversion), an elevation in GC content would result [92]. The double-strand breaks are preferentially repaired by sister chromatids, not the homologs [93]. Theoretically, the homeologs may have even smaller chance to be taken as repairing substrates for often sharing less sequence similarity. There is indirect evidence that nonallelic gene conversion can be related to GC changes in vertebrates [94–96]. As to the possible contribution from homeologous gene conversion, there have been some initial exploration in rice and sorghum [97,98]. GC elevation has been particularly prominent in grasses, resulting in two distinct gene classes with average GC content ∼50%, and ∼90% [97,98]. No correlation was found between gene conversion and GC content in rice and sorghum [30], with converted paralogs usually having similar GC content to non-converted paralogs (in rice: 0.58 *versus* 0.58; in sorghum: 0.58 *versus* 0.59). There is significantly higher GC content in the converted genes (average GC3 ∼0.76) than the non-converted ones (∼0.69). However, this small difference could not account for the evolution of two distinct groups of genes. If conversion increased GC content, we would find significant GC increase in the converted genes. In the region of chromosomes 11 and 12 where conversion may have recursively occurred and is possibly still on-going, GC elevation was not found, with genes in that region actually showing lower GC content (0.65) than the average of all collinear genes (0.71). This implies that ectopic gene conversion alone may not contribute to GC elevation. The correlation detected between GC content and conversion may perhaps be explained by higher GC content leading to higher sequence similarity. Taking an extreme example, in a random sequence comprised of only G and C, 50% of nucleotides can match merely by chance, *versus* 25% in two random sequences equally sampled from all four types of nucleotides. Many other factors have been related to GC enrichment, such as transcription, translation, methylation and mutation bias [99,100], making it still a topic of continuing interest.

## 6. Conclusions

The importance of ectopic gene conversion in plants may be generally underestimated. As one direct result of homoeologous recombination, gene conversion may affect the evolution of genes in large families, and those in syntenic positions on paleo-duplicated chromosomes. Recursive whole genome duplication has produced thousands of syntenic paralogs, which may convert one another at relatively high frequency initially but declining over time, contributing to intragenomic homogeneity and inter-genomic divergence. Though most genes may escape conversion by their homologs, concerted evolution of duplicated genes can last for millions of years or longer, which may buffer the establishment of novel gene functions elsewhere in a genome. A mixed evolutionary model, based on a primary birth-and-death process with occasional homoeologous recombination and gene conversion between duplicated genes, may best explain the evolution of multigene families.
